# Deciphering the Molecular Crosstalk of Endoplasmic Reticulum Stress and Immune Infiltration in Endometriosis

**DOI:** 10.1111/aji.70092

**Published:** 2025-05-15

**Authors:** Yuan Ma, Chunfang Ha, Ruyue Li, Ruiqi Zhang, Min Li

**Affiliations:** ^1^ Ningxia Medical University Yinchuan China; ^2^ General Hospital of Ningxia Medical University Yinchuan Ningxia China; ^3^ Key Laboratory of Fertility Preservation & Maintenance of Ministry of Education, Ningxia Medical University Yinchuan Ningxia China

**Keywords:** endometriosis, endoplasmic reticulum stress, immune system infiltration

## Abstract

**Background:**

Endometriosis (EMs), characterized by ectopic endometrial growth causing infertility. Endoplasmic reticulum stress (ERS) is an important cellular defense mechanism. However, the correlation between ERS and EMs remains unclear. We aimed to investigate the relationship between them, identify biomarkers, and offer new insights into the treatment of EMs.

**Methods:**

Two RNA expression datasets (GSE120103 and GSE25628) related to EMs in *Homo sapiens* were used to identify ERS‐differentially expressed genes (ERS‐DEGs). Protein–protein interaction (PPI) networks and CytoHubba (Cytoscape) identified ERS‐associated HUB genes, with receiver operating characteristic curves (ROC) evaluating diagnostic value. Constructed mRNA‐microRNA (miRNA)/RNA‐transcription factor (TF) interaction networks and performed ssGSEA to compare immune infiltration between EMs patients and controls. Real‐time quantitative polymerase chain reaction (RT‐qPCR), western blotting (WB), and immunohistochemistry (IHC) were performed to assess potential biomarker levels.

**Results:**

Thirty‐three ERS‐DEGs were identified, with nine HUB genes (*HSPA5, XBP1, HSP90B1, DNAJC3, PDIA3, PDIA6, PDIA4, HERPUD1*, and *MANF*) demonstrating diagnostic efficacy (AUC > 0.7). Furthermore, immune infiltration revealed a significant relationship between immune cell abundance and HUB gene expression. Experimental validation confirmed the consistency of four biomarkers (*HSPA5, HSP90B1, PDIA6*, and *HERPUD1*). Regulatory network analysis identified 62 miRNAs and 44 TFs interacting with HUB genes, suggesting a multifactorial immunometabolic axis.

**Conclusions:**

We identified four biomarkers (*HSPA5, HSP90B1, PDIA6*, and *HERPUD1*) associated with ERS that offer new insights into the detection and treatment of EMs. Our findings indicate an abnormal response to ERS and immune system infiltration contribute to the progression of EMs.

## 1 Introduction

Endometriosis (EMs) is a chronic inflammatory and estrogen‐dependent disease characterized by chronic pelvic pain and infertility [1]. It shows similarities in tumor characteristics in terms of invasion, migration, and distant growth, and it may transform into ovarian clear cell carcinoma or endometrioid carcinoma [2]. The endoplasmic reticulum (ER) is an organelle found in various eukaryotic cells and is responsible for protein synthesis, post‐translational modification, folding, and transportation. ER stress (ERS), also referred to as the unfolded protein response (UPR), serves as a cellular protective measure, eliminating misfolded proteins through the ER‐associated protein degradation (ERAD) system and recycling misfolded proteins through the induction of autophagy. In severe ERS and the inability to re‐establish ER homeostasis, the response triggers cell suicide or apoptosis [3, 4]. In EMs cells, autophagy and apoptosis rates are minimal. Studies [5, 6] have shown that in EMs, the UPR is activated and ERS proteins such as *GRP78* are upregulated to reduce apoptosis in ectopic endometrial stromal cells. ERS may promote neoangiogenesis and cell survival through the IL‐8 pathway [7], and it can also regulate the invasiveness of ectopic endometrial stromal cells by inhibiting the AKT/mTOR pathway [8]. UPR‐induced apoptosis can be inhibited by estrogen [9]. Guzel et al. [10] showed that tunicamycin, a potent ERS inducer, can suppress cell growth and promote apoptosis in human endometrial glandular and stromal cells. In conclusion, the occurrence of EMs is closely related to ERS.

Additionally, EMs, characterized by inflammation, promotes cell survival and attachment by introducing inflammatory factors into the abdominal microenvironment and ectopic tissues [11]. ERS can affect the inflammatory response through multiple mechanisms. ERS can activate the nuclear factor‐kB (NF‐κB) signaling pathway, thereby inducing an inflammatory response [12]. *GRP78* and *AKT* levels are elevated in ectopic tissues, and *GRP78* expression positively correlates with *AKT* expression. This suggests that ERS and PI3K/AKT signaling collectively contribute to the pathogenesis of EMs [13].

Considering that ERS may be an important mechanism mediating EMs pathogenesis, this study compared ERS‐related gene (ERSRG) expression differences in transcriptome sequencing data centers of healthy endometrium and abnormal lesions in EMs subjects in publicly available databases. Machine learning techniques were applied to select features to identify possible diagnostic markers and study their underlying mechanisms and immune milieu. These findings provide insight into the molecular pathways for diagnosing EMs in connection with ERS and immune responses.

## 2 Materials and Methods

### 2.1 Data Download and Differential Analysis

We obtained valuable information by accessing the datasets GSE120103 and GSE25628 related to *Homo sapiens* from the GEO repository [14] using the R package GEOquery [15]. The dataset GSE120103 comprised 36 samples, including 18 EMs and 18 healthy control samples. Similarly, the GSE25628 dataset contained 13 samples: seven EMs and six healthy control samples (Table S1). The GSE120103 and GSE25628 datasets were combined into a single, merged, and standardized dataset. Subsequently, batch correction of the integrated dataset was performed using the R package sva [16], and then the limma package was used to obtain differentially expressed genes (DEGs) between the EMs and control groups.

By searching the keyword “Endoplasmic reticulum stress,” we identified 10 746 ERSRGs in the GeneCards database. The MSigDB database contained 272 ERSRGs [17]. Combining ERSRGs from both sources resulted in a total of 240 genes (Table S2).

We then performed |gap analysis logFC| > 1 and P.adjust < 0.05, DEGs and ERSRGs get intersection and map Wayne, get ERS‐DEGs), and through the R package ggplot2 map, volcanic, and heatmaps present the results of the variance analysis.

### 2.2 Gene Ontology (GO) and Kyoto Encyclopedia of Genes and Genomes (KEGG) Enrichment Analysis

GO [18] is a prevalent technique for extensive functional enrichment investigations, covering biological processes (BPs), molecular functions (MFs), and cellular components (CCs). The KEGG is an extensively used repository that contains details on genomes, biological pathways, diseases, and medications. To conduct GO annotation analysis of ERS‐DEGs, we used the R software package clusterProfiler. The screening criteria for entry were P.adjusted < 0.05 and FDR value (*q*‐value) < 0.05.

### 2.3 Gene Set Enrichment Analysis (GSEA)

GSEA [19] is a computational technique developed by the Broad Institute that identifies whether a predefined set of genes exhibits significant differences between two biological conditions. It is widely used to assess alterations in pathway activity and BPs in expression data samples. To explore variations in BPs among the sample groups, we obtained the reference gene set “c2.cp.v7.2.symbols.gmt” from the MSigDB database [20]. The GSEA method integrated into the R package was employed to analyze and visualize the dataset. The parameters used in GSEA included setting the seed to 2020, conducting 1000 computations, and ensuring that each gene set contained a minimum of 10 genes and a maximum of 500 genes. The Benjamini–Hochberg method was used for *p* value correction. The criteria for identifying significant enrichment were set as p.adjust < 0.05 and FDR value (*q* value) < 0.05.

### 2.4 Gene Screening and Validation of HUB Genes Associated With ERS in EMs

Genes associated with ERS were analyzed using the STRING database and a protein interaction network was constructed. After visualization, HUB genes were selected according to the CytoHubba plugins closeness centrality (closeness), degree, edge‐percolated component (EPC), maximal clique centrality (MCC), and maximum neighborhood component (MNC), and an interactive network of HUB genes was derived using the GeneMANIA [21] website. Additionally, we evaluated the diagnostic value of these HUB genes using receiver operating characteristic (ROC) [22] curves and verified their expression by quantitative polymerase chain reaction (qPCR), western blotting (wb), and immunohistochemistry (IHC).

### 2.5 Construction of mRNA‐microRNA (miRNA) and mRNA‐transcription Factor (TF) Interaction Networks

miRNAs play a crucial role in regulating biological development and evolution, with the ability to control a wide range of target genes. Multiple miRNAs can regulate the expression of the same gene. The miRDB database [23] was used to predict the target genes and provide functional annotations of miRNAs. The prediction of miRNAs interacting with HUB genes was made utilizing the miRDB database, followed by the creation of an mRNA–miRNA interaction network based on a target score ≥ 85 criterion from the database. Additionally, the CHIPBase database [24] (version 3.0) uses ChIP‐seq data to identify DNA‐binding proteins and their associated binding sites and predicts numerous TFs and gene transcription regulatory relationships by combining the base sequence matrix. By searching the CHIPBase database for TF binding to HUB genes, data indicating the number of samples found (upstream) + number of samples found (downstream) ≥ 5 were selected to construct the mRNA‐TF interaction network.

### 2.6 Immune Infiltration Analysis

The evaluation of gene sets in a single sample, referred to as GSEA using a single sample (ssGSEA), assessed the infiltration ratio of each specific immune cell. Initially, all types of infiltrating immune cells were recognized, including activated CD8 T cells, activated dendritic cells, gamma delta T cells, natural killer cells, and various other human immune cell subgroups, such as regulatory T cells. Subsequently, the enrichment scores derived from ssGSEA were utilized to represent the percentage of immune cell infiltration in individual samples, resulting in the creation of an immune cell infiltration matrix. The ggplot2 software package was used to generate visual comparisons between groups based on ssGSEA immune infiltration analysis, highlighting the varied expression results in the dataset. Additionally, a heatmap software package was used to produce correlation heatmaps that exhibited both the immune infiltration matrix and significant connections between immune cells. Finally, a correlation heatmap was designed to analyze the relationships between HUB genes and ssGSEA immune cells present in the dataset.

### 2.7 Sample Sources for Real‐Time qPCR (RT‐qPCR) Validation

The study included nine cases of ectopic endometriotic lesions and nine cases of matched normal endometrium. These patients underwent hysteroscopy–laparoscopic surgery at the General Hospital of Ningxia Medical University between January 2023 and December 2023. Their age ranged from between 25 and 35 years. The diagnosis of ovarian EMs cysts was confirmed in the experimental group based on the findings during surgery and subsequent pathological examination. The control group consisted of nine individuals with tubal infertility who underwent hysteroscopy–laparoscopic surgery during the same period, all showing a normal endometrium. Intraoperative and postoperative assessments did not reveal any instances of EMs, uterine fibroids, uterine polyps, endometrial cancer, or other similar conditions. The age range of these individuals was 25–35 years, with no significant difference between the two groups (*p* > 0.05). To ensure the credibility of the study, none of the participants had used any hormone medications in the 3 months leading up to their surgeries. This study was approved by the ethics committee of the General Hospital of Ningxia Medical University (ethics number: KYLL‐2024‐1486), and all participants and their families provided informed consent. The collected samples were quickly frozen in liquid nitrogen within 30 min of removal and preserved at −80°C for further examination.

### 2.8 RNA Extraction and RT‐qPCR

RNA was extracted from nine normal endometrial and nine EC samples following the protocol provided by ABP Biosciences Inc., utilizing Nuclezol LS RNA Isolation Reagent. Subsequently, total RNA was transcribed into cDNA using the SureScript‐First‐strand‐cDNA‐synthesis‐kit from GeneCopoeia according to the manufacturer's guidelines. qPCR analysis was performed using BlazeTaq SYBR Green qPCR Mix 2.0 from GeneCopoeia. The thermal cycling parameters for qPCR included initial denaturation at 95°C for 30 s, followed by 40 cycles of denaturation at 95°C for 10 s, annealing at 60°C for 20 s, and extension at 72°C for 30 s expression levels were normalized using the endogenous control GAPDH and analyzed using the 2−DDCq method. Statistical comparisons between groups were performed using the *t*‐test, with statistical significance set at a two‐tailed *p* value < 0.05.

### 2.9 Protein Extraction and WB Assay

Total protein was extracted from three normal endometrial and three EC samples using RIPA lysis buffer (PC101; EpiZyme, Shanghai, China). The total protein concentration was measured using a BCA protein assay (WB6501; NCM Biotech, Suzhou, China). Equivalent amounts of proteins (25 µg) were separated by 10% SDS‐PAGE and transferred onto 0.45‐µm polyvinylidene fluoride membranes (IPVH00010; Millipore, Darmstadt, Germany). After blocking, the membranes were incubated with primary antibodies against HSPA5 (11587‐1‐AP, 1:6000, Wuhan, China), HERPUD1 (10813‐1‐AP, 1:750), HSP90B1 (14700‐1‐AP, 1:4000), DNAJC3 (26721‐1‐A, 1:750), PDIA6 (18233‐1‐AP, 1:2000), and GAPDH (60004‐1‐Ig, 1:10000) at 4°C overnight. The membranes were then incubated with secondary antibodies (SA00001‐2, 1:7500) at room temperature for 1 h. Finally, the protein bands of interest were analyzed and quantified using ImageJ software.

### 2.10 IHC Analysis of HUB Genes

IHC analysis was conducted on tissue sections derived from paraffin‐embedded blocks of nine EC samples and nine normal endometrial tissues. Antigen retrieval was performed using citrate buffer (pH 6.0) for heat‐induced epitope retrieval. The primary antibodies used were against *PDIA6* (1:800), *HSP90B1* (1:1500), *HERPUD1* (1:300), *DNAJC3* (1:1600), and *HSPA5* (1:1600). For *DNAJC3* and *HSPA5*, EDTA‐based antigen retrieval was employed. All primary antibodies were incubated overnight at 4°C, followed by detection with the appropriate horseradish peroxidase‐conjugated secondary antibodies. The chromogenic substrate 3,3ʹ‐diaminobenzidine was used to visualize antibody binding, and the slides were counterstained with hematoxylin. Negative controls were included by omitting primary antibodies to ensure the specificity of staining.

### 2.11 Statistical Analysis

Data processing and analysis were conducted using R software (Version 4.2.3). Continuous variables are presented as mean ± SD. The Wilcoxon rank‐sum test was used to compare continuous variables between two groups, whereas an independent Student's *t*‐test was used for normally distributed variables. For comparisons involving three or more groups, the Kruskal–Wallis test was used. Categorical variables were compared using the Chi‐square test or Fisher's exact test. ROC curves were generated using the R package. Spearman's correlation analysis was used for unspecified results, with all two‐sided *p* values. Statistical significance was set at *p* < 0.05.

## 3 Results

### 3.1 Identification of ERS‐DEGs Associated With EMs

**FIGURE 1:**
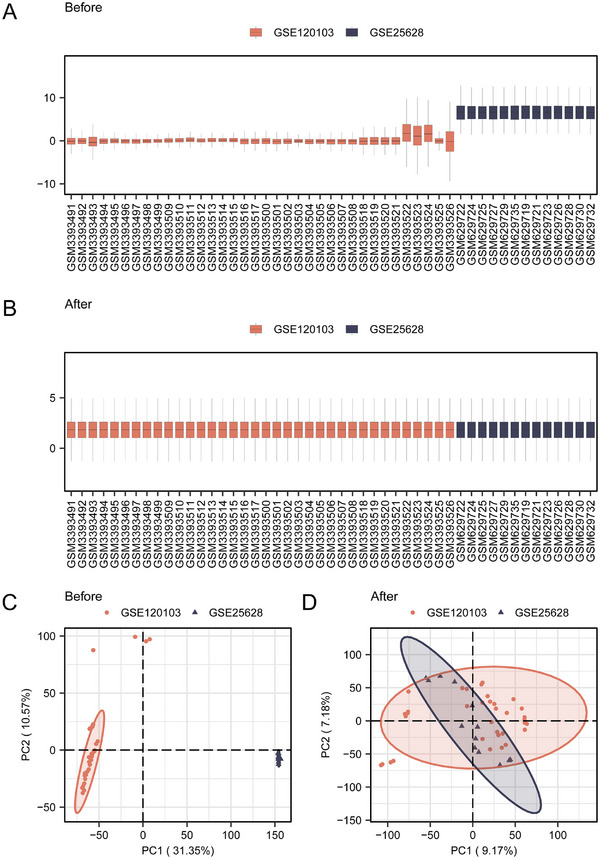
Screening of DEGs associated with EMs. (A) Boxplot of the merged dataset before dataset correction. (B) Boxplot after correction of the merged dataset. (C) PCA plot of the combined dataset before correction. (D) PCA plot after correction of the combined dataset.

**FIGURE 2:**
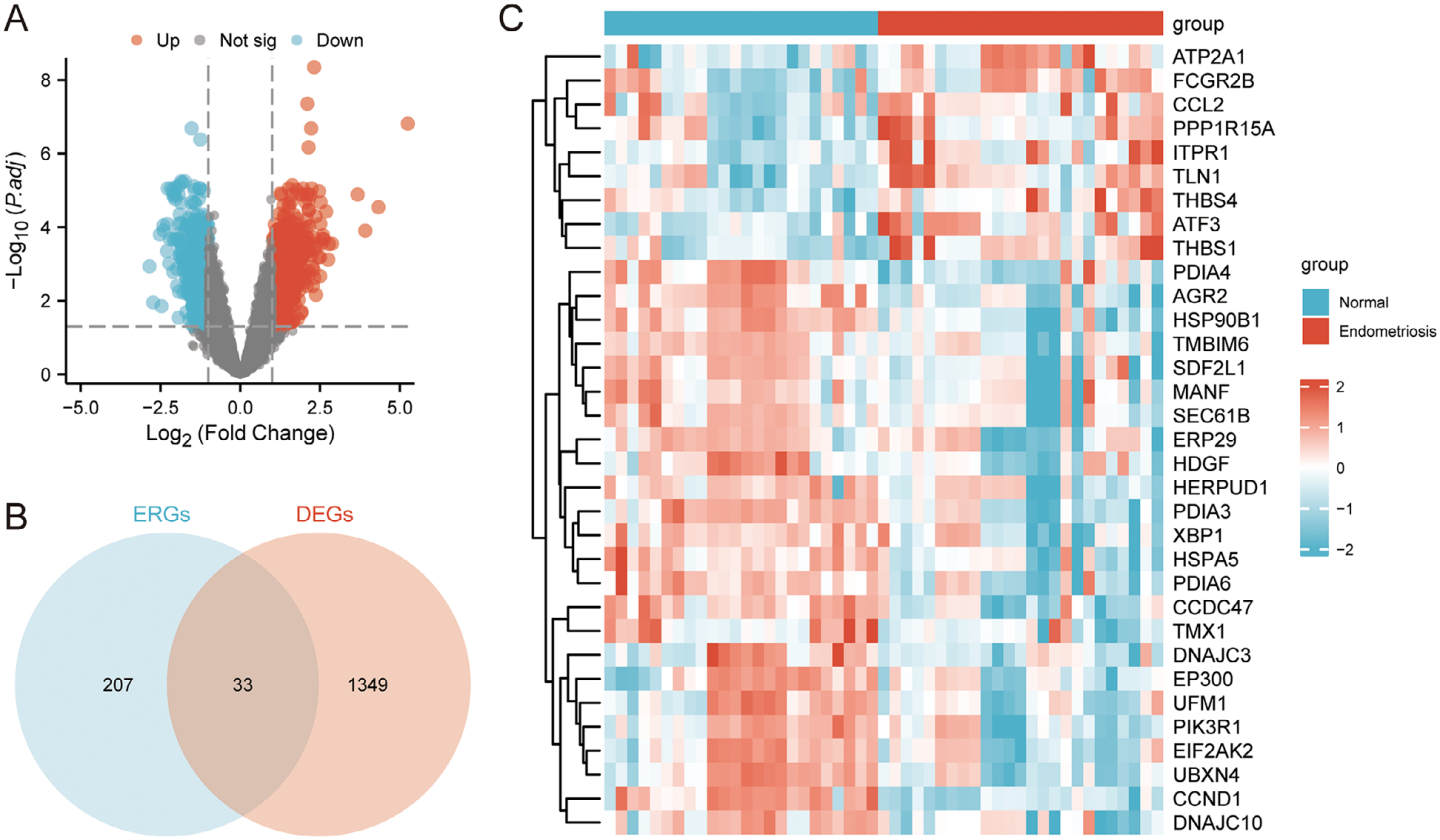
Identification of ERSRGs associated genes in EMs. (A) Volcano plot of DEG analysis between the normal and EMs groups in the combined dataset. (B) Venn diagram of DEGs and ERSRGs in the combined dataset. (C) Heatmap of ERS‐DEGs in the combined dataset. DEGs, differentially expressed genes; ERSRGs, ERS‐related genes.

By combining the two selected datasets (Figure 1A–D), 1382 DEGs were obtained by differential gene analysis, including 734 upregulated and 648 downregulated genes (Figure 2A). Thirty‐three ERS‐DEGs were obtained by taking the intersection of 1382 DEGs with 207 ERSRGs (Figure 2B), including *AGR2, ATF3, ATP2A1, CCDC47, CCL2, CCND1, DNAJC10, DNAJC3, EIF2AK2, EP300, ERP29, FCGR2B, HDGF, HERPUD1, HSP90B1, HSPA5, ITPR1, MANF, PDIA3, PDIA4, PDIA6, PIK3R1, PPP1R15A, SDF2L1, SEC61B, THBS1, THBS4, TLN1, TMBIM6, TMX1, UBXN4, UFM1*, and *XBP1*. The significantly different expression of these ERS‐DEGs between the EMs and normal groups was visualized using a heatmap (Figure 2C).

### 3.2 Functional and Signaling Pathway Enrichment of 33 ERS‐DEGs

**FIGURE 3:**
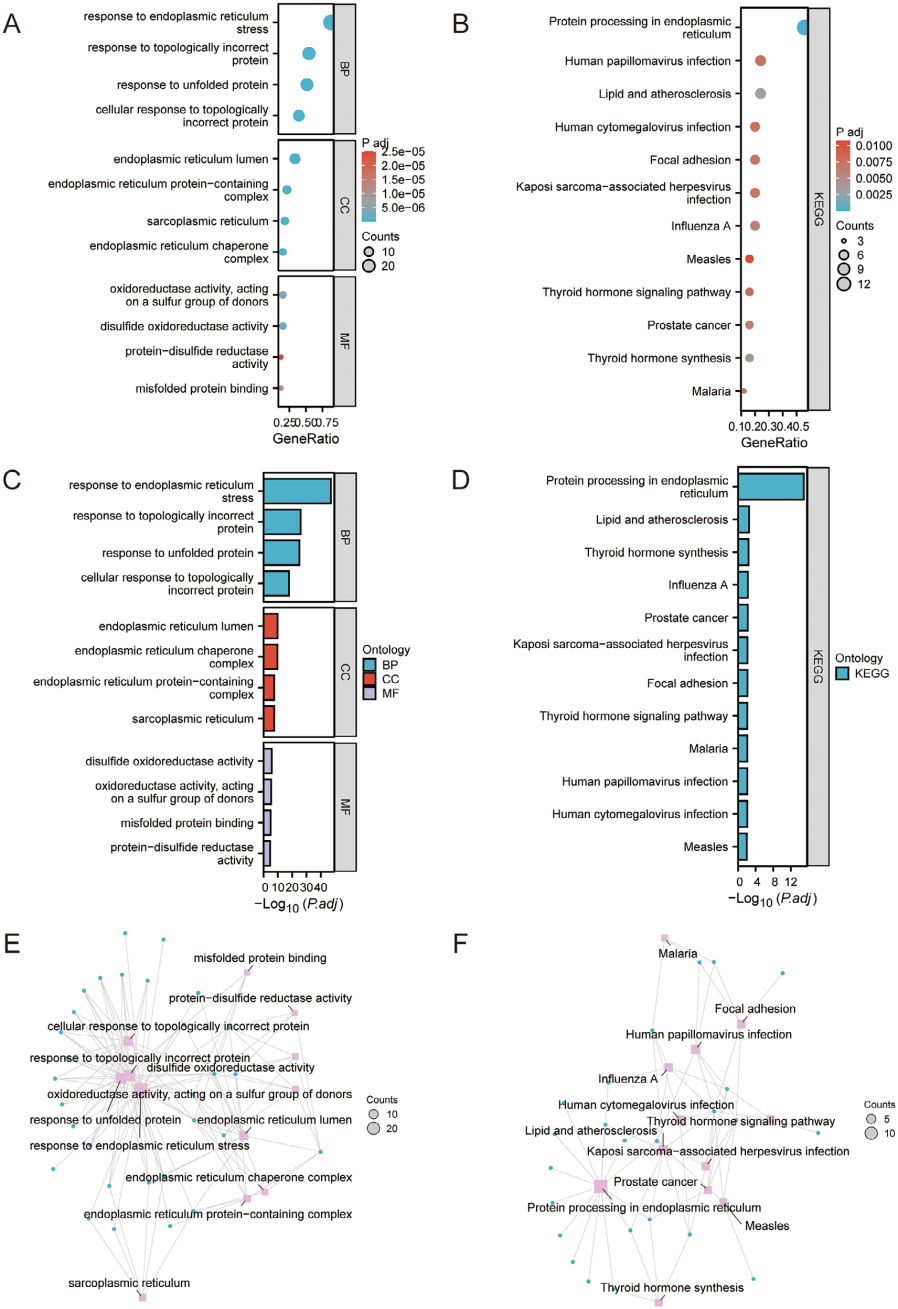
Functional and signaling pathway enrichment of 33 ERS‐DEGs. (A) Bubble plot of GO enrichment analysis. (B) Bubble plot of KEGG enrichment analysis. (C) Bar chart of GO enrichment analysis. (D) Bar chart of KEGG enrichment analysis. (E) Network diagram of GO functional enrichment analysis of CSDEGs. (F) Network diagram of KEGG pathway enrichment analysis of CSDEGs. The screening criteria for significantly enriched GO and KEGG entries were p.adjust < 0.05 and FDR value (*q*‐value) < 0.05. GO, gene ontology; BP, biological process; CC, cellular component; MF, molecular function; KEGG, Kyoto Encyclopedia of Genes and Genomes.

GO annotation revealed that terms associated with ERS were enriched for BPs, CCs, and MFs. The analysis showed that in BPs, protein processing in the ER and response to topologically incorrect proteins, together with ERS function, were enriched, indicating that ERS activation may be an important factor in EMs pathogenesis. In CCs, ERS‐DEGs were mainly associated with the ER lumen, sarcoplasmic reticulum, and ER protein‐containing complex. In MFs, ERS and oxidoreductase activity were significantly enriched. Additionally, KEGG analysis showed that ERS‐DEGs mainly participated in lipid and atherosclerosis, thyroid hormone synthesis, focal adhesion, viral infection, and other routes (Figure 3A–F, Tables S3 and S4).

### 3.3 GSEA ERS‐DEGs in EMs

**FIGURE 4:**
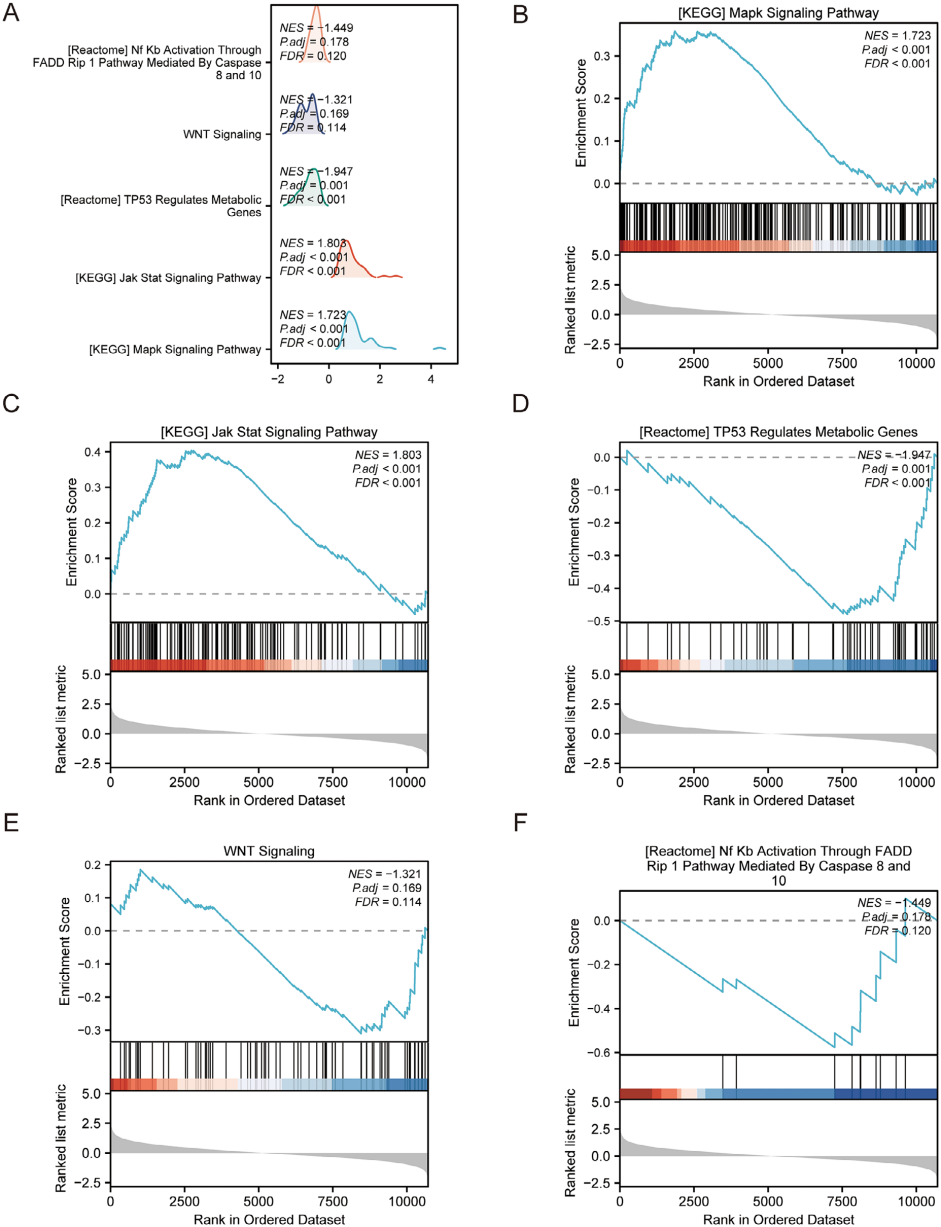
Gene set enrichment analysis (GSEA) of 33 ERS‐DEGs. (A) GSEA of the EMs/normal group samples in the combined dataset showed four main biological characteristics. (B–E) MAPK (B) and JAK‐STAT signaling pathways (C). TP53 regulates metabolic genes (D), Wnt signaling (E), and NF‐κB activation through the FADD Rip 1 pathway mediated by caspases 8 and 10 (F) and other pathways.

The effects of ERS‐DEG levels on the occurrence of EMs and on the correlation between the expression of these genes and BPs, CCs, and MFs were evaluated. GSEA revealed significant enrichment of ERS‐DEGs in multiple biological pathways implicated in key BPs, such as signaling, metabolic regulation, immune response, and cell cycle control. The MAPK signaling pathway, JAK‐STAT signaling pathway, metabolic genes regulated by TP53, Wnt signaling pathway (Figure 4A–E), and NF‐κB are important TFs involved in immune response and cell survival (Figure 4F). GSEA revealed the following pathways: neuroactive ligand–receptor interaction, cytokine–cytokine receptor interaction, G protein‐coupled receptor class A rhodopsin‐like, and acute inflammatory and profibrotic mediators (Table S5). These findings provide new insights into the role of ERS in EMs and may provide potential targets for EMs diagnosis and treatment.

### 3.4 HUB Genes Related to ERS of EMs: Identification, Interactions, and Diagnostic Potential Evaluation

**FIGURE 5:**
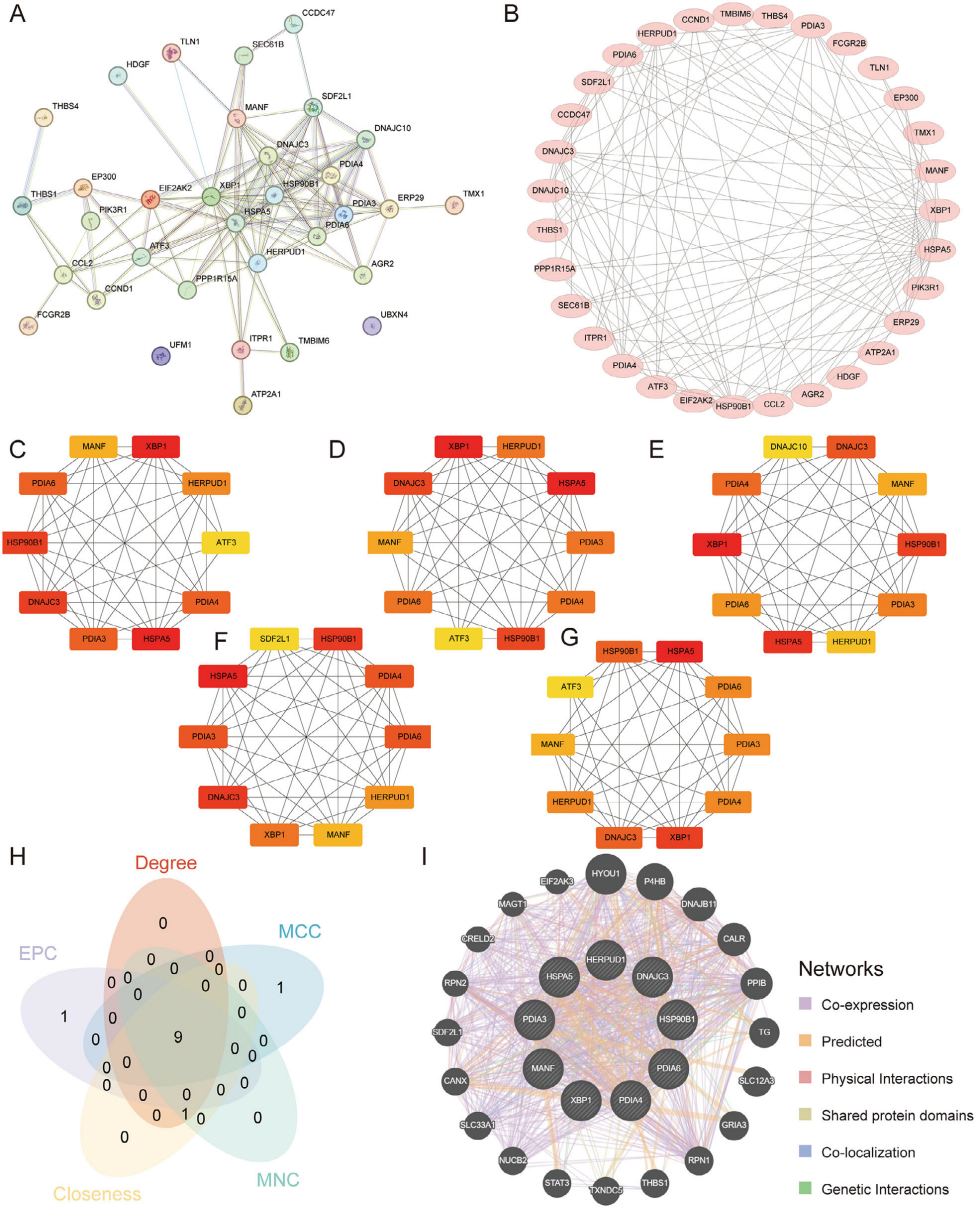
Screening HUB genes associated with ERSRGs in EMs. (A) PPI network of the STRING database. (B) PPI network of the Cytoscape database. (C–G) PPI network of HUB genes under the Closenes (C), Degree (D), EPC (E), MCC (F), and MNC (G) algorithms; colors from light to dark represent scores from low to high. H. closeness, degree, EPC, MCC, and MNC five‐algorithm Venn diagram. I. The reciprocal network was collected and derived from the GeneMANIA website, where black circles with white slash lines represent input HUB genes, and other black circles without white slash lines represent functionally predicted genes. The red line represents the physical interactions between genes, purple line represents the co‐expression between genes, yellow lines represent shared protein domains between genes, blue lines represent co‐localization relationships between genes, and green lines represent genetic interactions between genes. ERS‐DEGs, ERS‐differentially expressed genes; PPI, protein–protein interaction; Degree, degree correlation; MNC, maximum neighborhood component; MCC, maximal clique centrality; EPC, edge‐percolated component; Closeness, closeness centrality.

Thirty‐three ERS‐DEGs were analyzed by protein–protein interaction (PPI) based on the top 10 genes identified by various algorithms and nine HUB genes (*HSPA5, XBP1, HSP90B1, DNAJC3, PDIA3, PDIA6, PDIA4, HERPUD1*, and *MANF*), including observed interactions, co‐expression patterns, and co‐localization trends. The results showed that *XBP1, DNAJC3, PDIA3/4/6*, and *HSPA5* were all involved in the ERS response and may be involved in protein folding, repair, and degradation to maintain protein homeostasis. *HSPA5* and *HSP90B1* play a protective role in response to cellular stress (e.g., heat, oxidative stress, and inflammation). The role of these genes in response to stress is to modulate immune responses, control the cell cycle, and participate in signaling (Figure 5A–I).

**FIGURE 6:**
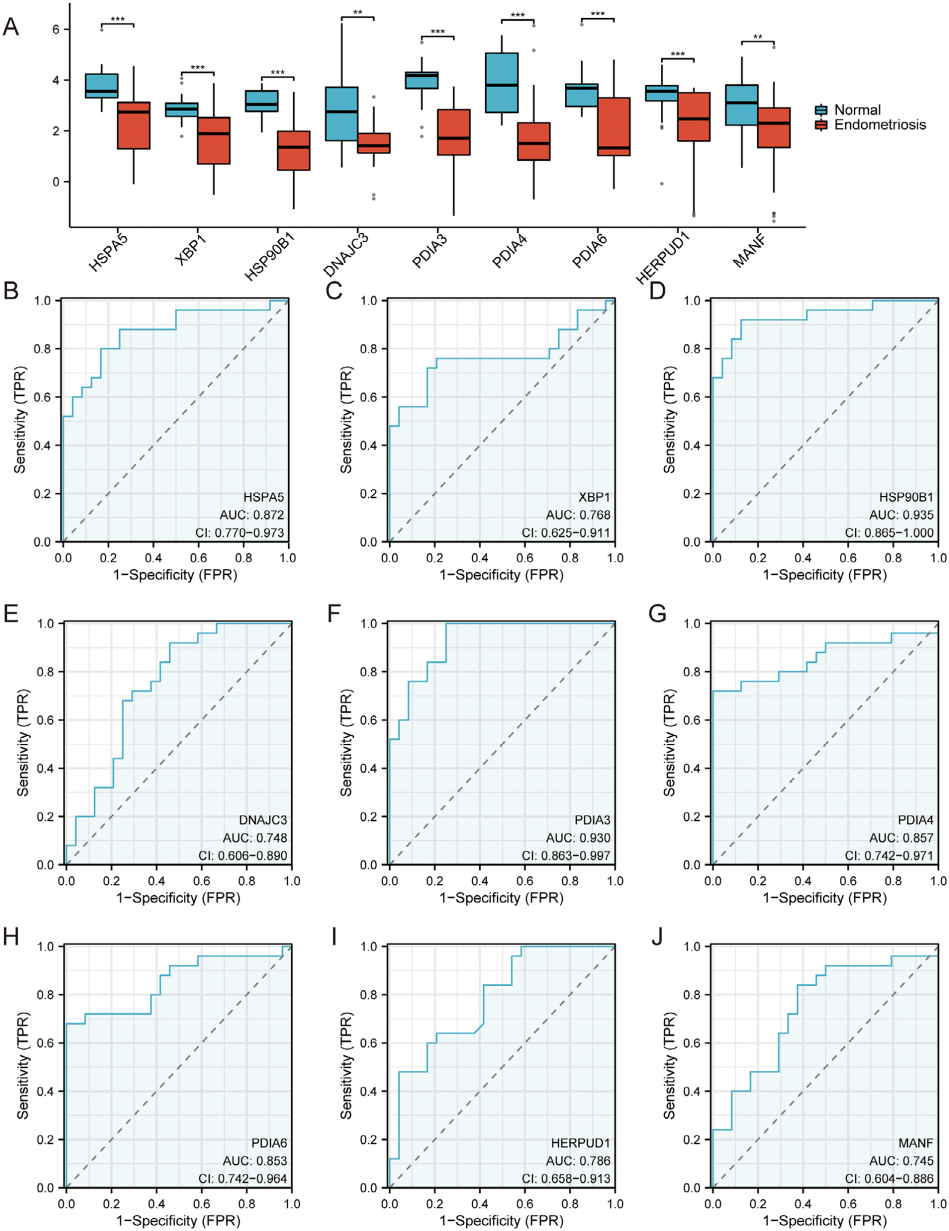
Group comparison plot and ROC curve. (A) Group comparison graph shows the difference in the expression of HUB genes in the dataset. Statistical methods: Mann–Whitney *U* test (Wilcoxon rank‐sum test). (B–E) ROC curve of nine HUB genes (*HSPA5, XBP1, HSP90B1, DNAJC3, PDIA3, PDIA4, PDIA6, HERPUD1*, and *MANF*) with normal and EMs as outcome variables. The symbol * is equivalent to *p* < 0.05, which is statistically significant, and ** represents *p* < 0.01, *** represents *p* < 0.001. The area under the ROC curve values are generally between 0.5 and 1. The closer the AUC is to 1, the better is the diagnostic performance. When the AUC was between 0.5 and 0.7, the accuracy was low; when the AUC was between 0.7 and 0.9, the accuracy was moderate; and when the AUC was above 0.9, the accuracy was high. FPR, false positive rate; TPR, true positive rate; ROC, receiver operating characteristics.

Further analysis of these HUB genes using the Mann–Whitney *U* test revealed significant differences in expression levels between the normal and EMs groups. The relationship between these HUB genes and EMs was further explored through ROC curve analysis, which demonstrated the potential of the genes as predictive biomarkers for EMs, with *HSP90B1, HSPA5*, and *PDIA3* exhibiting particularly strong predictive capabilities, as evidenced by AUC values > 0.9 (Figure 6A–J, Table S6). This study underscores the significant role of these HUB genes in the pathophysiology of EMs and suggests their potential use in diagnostic and therapeutic strategies.

### 3.5 Analysis of miRNA and TF Regulatory Networks for ERS‐Related HUB Genes

**FIGURE 7:**
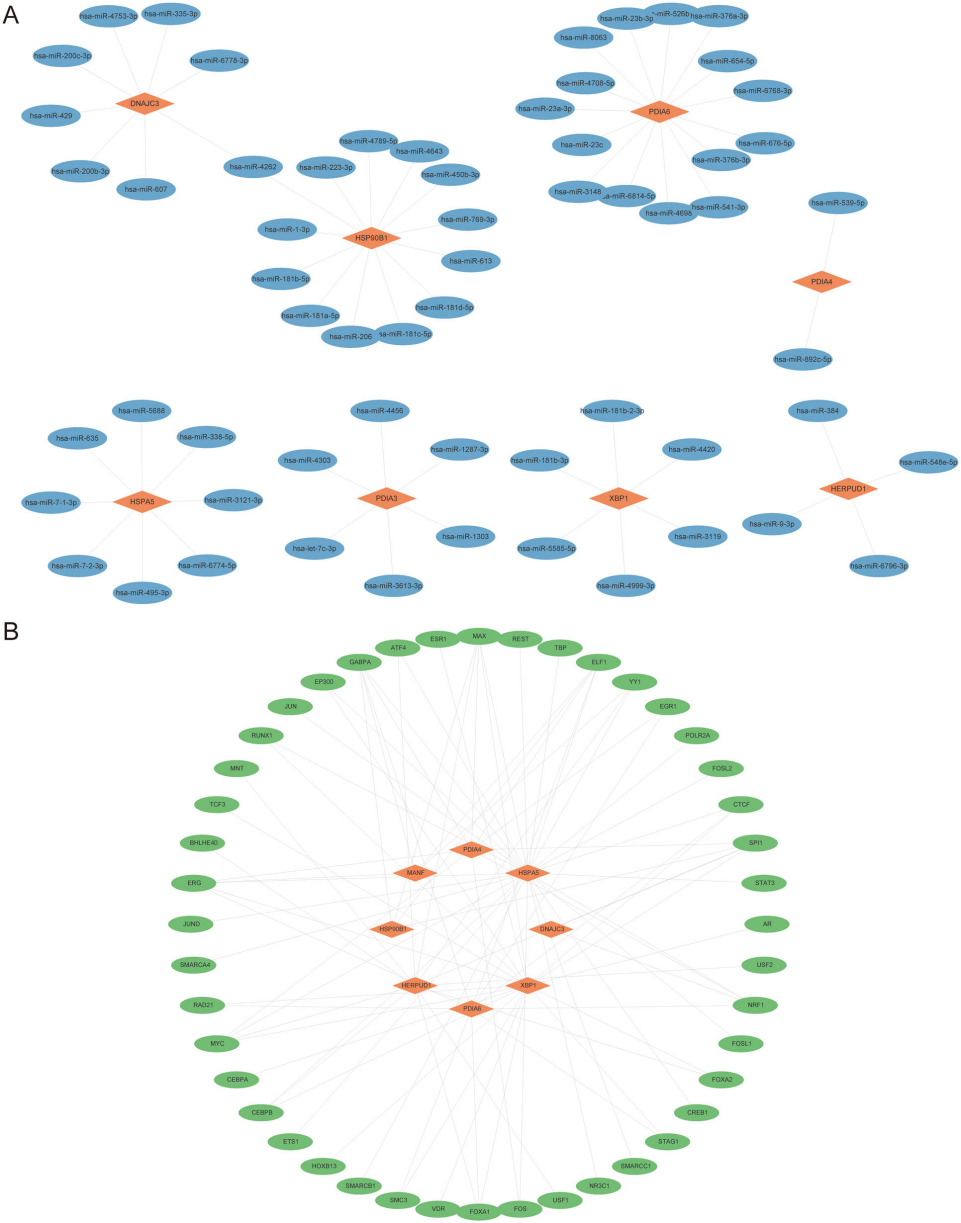
mRNA‐miRNA and mRNA‐TF interaction networks of HUB genes. (A) mRNA‐miRNA interaction network of HUB genes. (B) mRNA‐TF interaction network of HUB genes. The orange diamonds in the mRNA‐miRNA (A) interaction network represent mRNAs and the blue ovals represent miRNAs. The orange diamonds in the mRNA‐TF (B) interaction network represent mRNAs and the green ovals represent transcription factors. TF, transcription factor.

In this study, the potential interactions of nine HUB genes with miRNAs were analyzed using the miRDB database. miRNAs with a target score greater than 90 were selected and visualized using the software (Figure 7A, Table S7). The analysis revealed a complex mRNA‐miRNA interaction network containing eight central genes and 62 miRNAs. TFs play key roles in post‐transcriptional regulation of gene expression by interacting with target genes (mRNAs). By exploring the TF target database, we identified TFs bound to HUB genes, including eight HUB genes and 44 TFs, forming 87 mRNA‐TF interaction relationships. These interactions were visualized using Cytoscape software (Figure 7B, Table S8). Based on these results, it can be seen that miRNAs and TFs play important roles in regulating HUB genes related to ERS, and these regulatory relationships may be important in the development and progression of EMs.

### 3.6 Analysis of the Relationship Between Immune Cell Infiltration and HUB Genes in EMs

**FIGURE 8:**
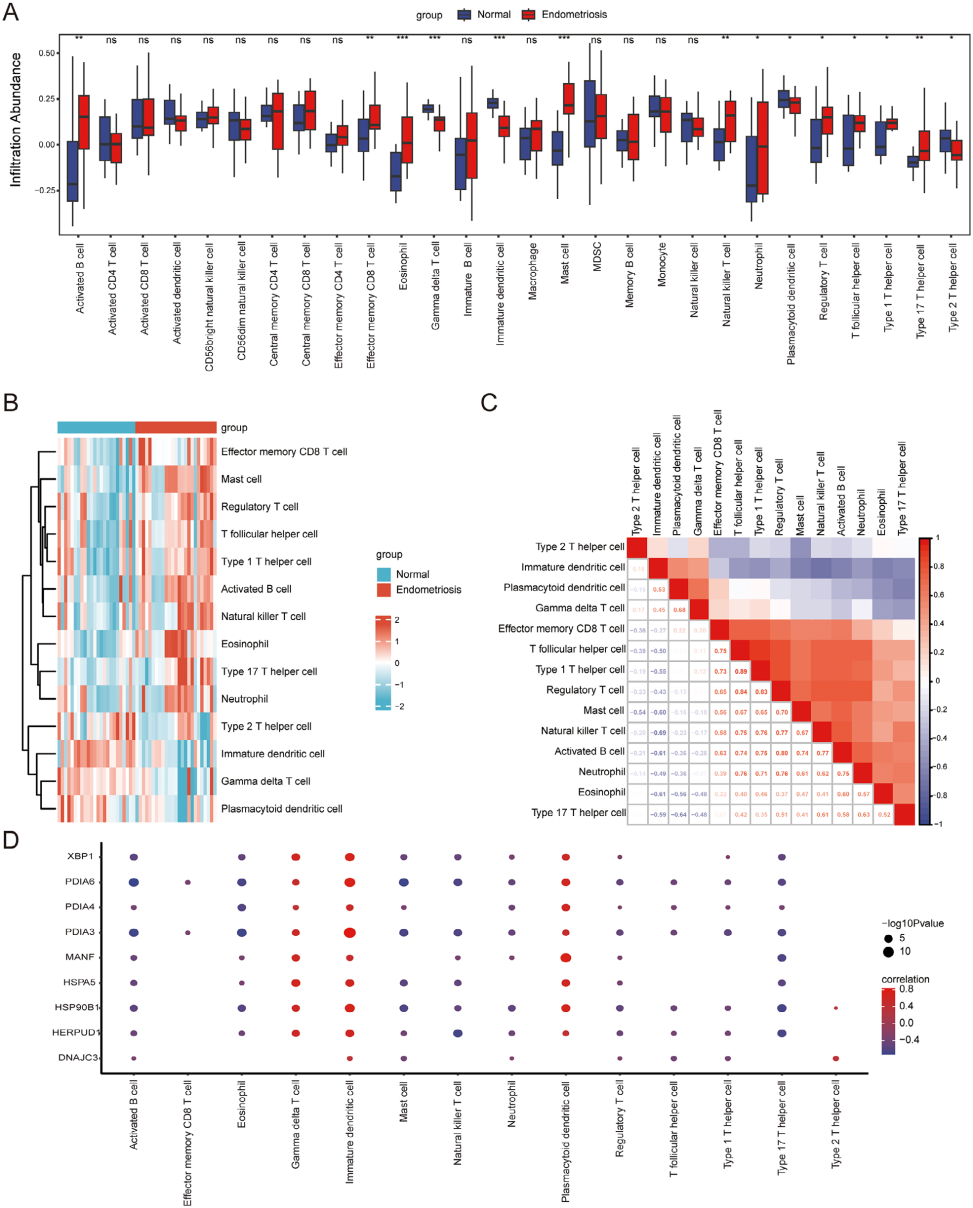
Immune cell infiltration analysis of dataset samples of HUB genes. (A) Group comparison diagram of ssGSEA immune infiltration analysis results for dataset samples. (B) Heatmap of ssGSEA immune infiltration analysis results for dataset samples. (C) The results of the correlation analysis of immune cell infiltration abundance of the dataset samples are presented. (D) Heatmap of the correlation between immune cell infiltration abundance and expression levels of nine HUB genes. The symbol ns is equivalent to *p* < 0.05, indicating no statistical significance. The symbol * is equivalent to *p* < 0.05, which is statistically significant. The symbol ** is equivalent to *p* < 0.01, which is highly statistically significant. The symbol *** is equivalent to *p* < 0.001, which is highly statistically significant.

Using the ssGSEA algorithm, we evaluated the infiltration levels of 28 immune cells in the normal and EMs groups. There were significant differences in the number of infiltrating cells between the two groups. In the EMs group, 14 immune cells were mainly associated with them, among which activated B cells, natural killer T cells, neutrophils, central memory CD4 T cells, and CD8T cells were positively associated with dysplasia (Figure 8A–C). Next, the correlation between immune cell infiltration abundance and expression levels of the nine HUB genes was analyzed. The nine HUB genes were clearly associated with plasmacytoid dendritic cells, immature dendritic cells, and gamma delta T cells. *PDIA6* and *PDIA3* correlated with almost all the 14 immune cells (Figure 8D). This suggests that the expression patterns of these genes may be related to the development, activation, and function of immune cells and their specific roles in the immune response.

### 3.7 EMs Diagnostic Potential: Verification of HUB Genes at the mRNA and Protein Levels

**FIGURE 9:**
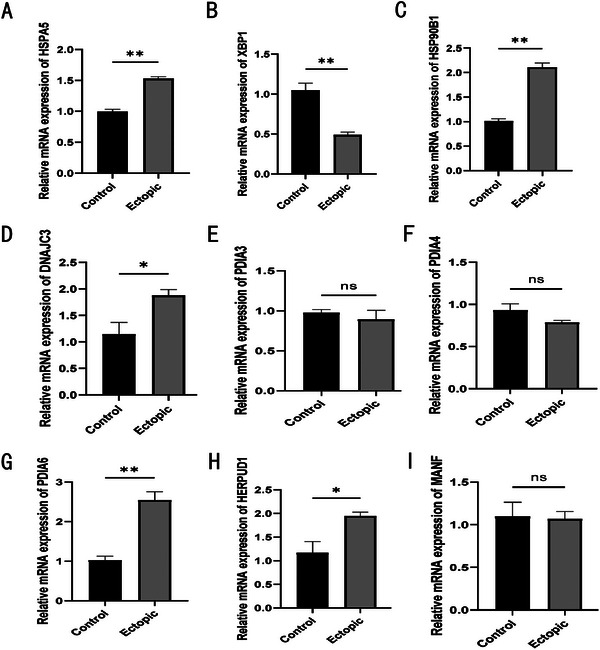
(A–I) qPCR results of nine HUB genes (*HSPA5, XBP1, HSP90B1, DNAJC3, PDIA3, PDIA4, PDIA6, HERPUD1*, and *MANF*) with normal and EMs as outcome variables. The symbol * is equivalent to *p* < 0.05, which is statistically significant, ** represents *p* < 0.01, which is highly statistically significant, and *** represents *p* < 0.001, which is highly statistically significant.

**FIGURE 10:**
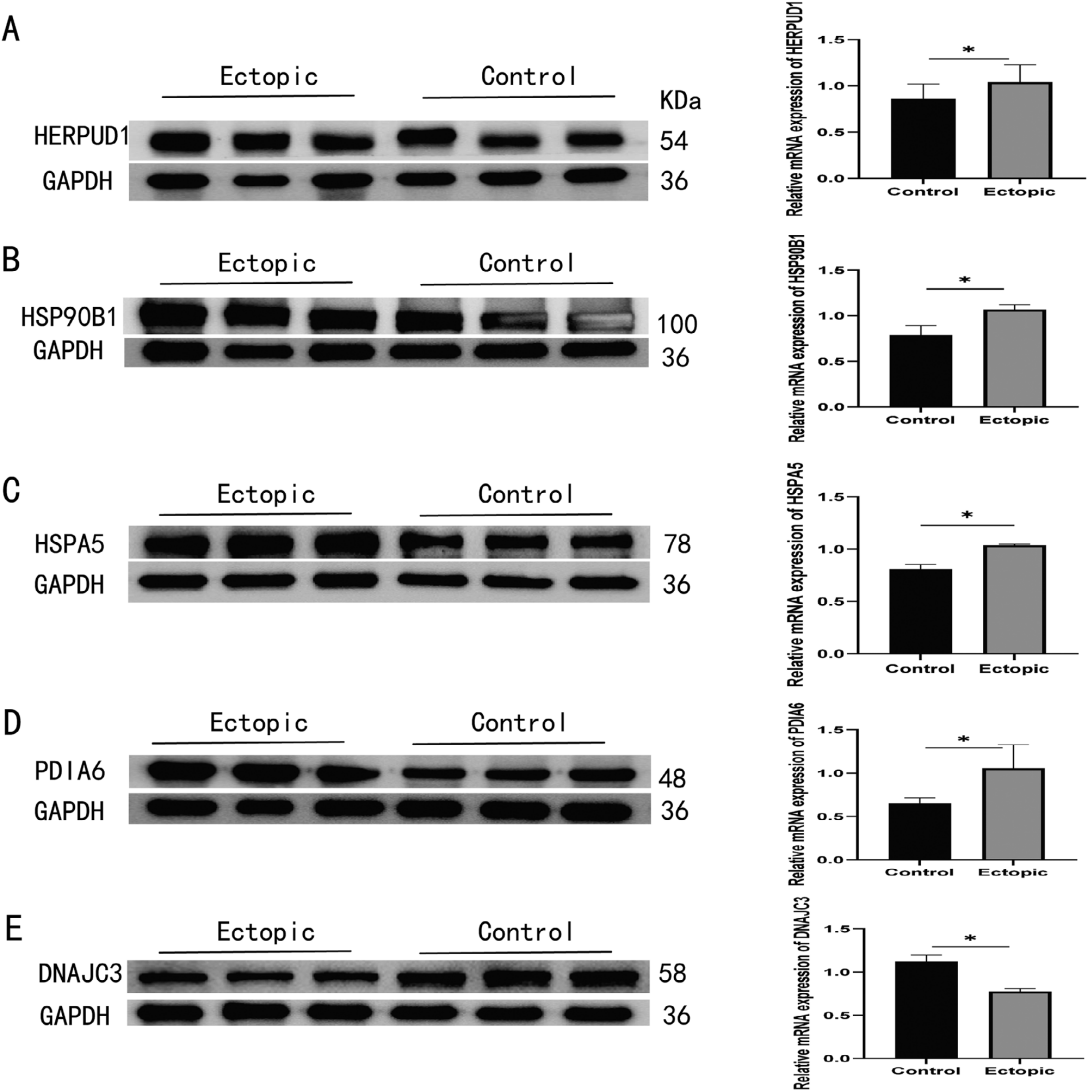
(A–E) WB results of five HUB genes (*HSPA5, HSP90B1, DNAJC3, PDIA6*, and *HERPUD1*) with normal and EMs as outcome variables. The symbol * is equivalent to *p* < 0.05, which is statistically significant, ** represents *p* < 0.01, which is highly statistically significant, and *** represents *p* < 0.001, which is highly statistically significant.

**FIGURE 11:**
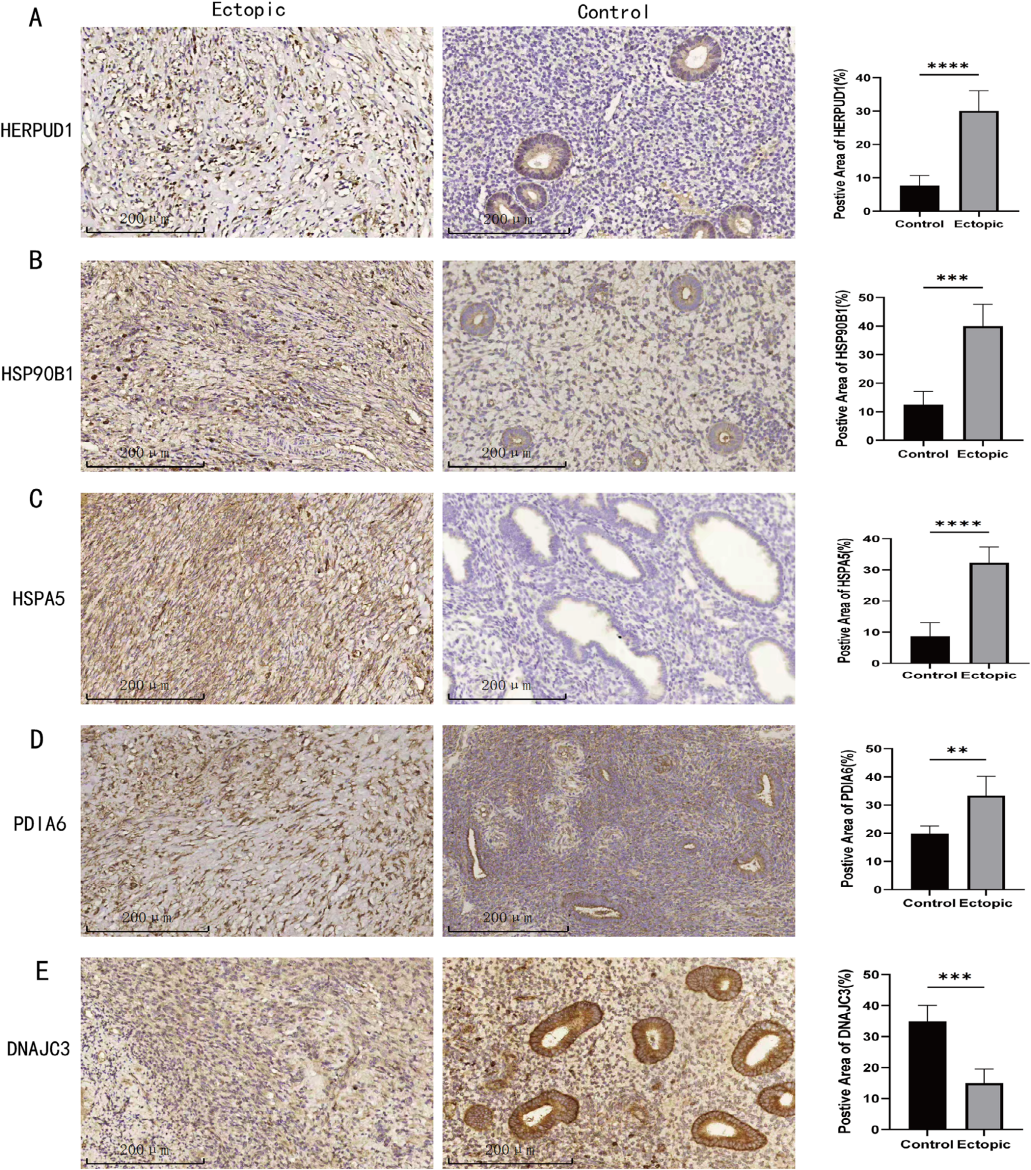
(A–E) IHC results of five HUB genes (*HSPA5, HSP90B1, DNAJC3, PDIA6*, and *HERPUD1*) with normal and EMs as outcome variables. The symbol * is equivalent to *p* < 0.05, which is statistically significant, ** represents *p* < 0.01, which is highly statistically significant, and *** represents *p* < 0.001, which is highly statistically significant.

The mRNA expression levels of nine HUB genes were verified by qPCR analysis of ovarian endometriotic cyst and normal ovarian tissue samples. We found that five genes, *HSPA5, HSP90B1, DNAJC3, PDIA6*, and *HERPUD1*, were significantly upregulated in ectopic tissues compared with normal tissues (Figure 9A–I). We then verified the protein expression of these genes using IHC and WB. Among the five genes identified through qPCR screening, four HUB genes (*HSPA5, HSP90B1, PDIA6*, and *HERPUD1*) showed significantly high protein expression levels in the EC group (Figures 10A–E and 11A–E).

This finding suggests that these four genes may serve as potential diagnostic biomarkers for EMs and may play key roles in pathophysiological processes such as protein homeostasis maintenance, cellular stress response, and immune regulation.

## 4 Discussion

EMs affects female fertility [1], but the exact etiology is unclear [25]. ERS is believed to be closely related to the development of EMs, which may affect cell growth and death and promote ectopic endometrial cell growth. As an inflammatory disease, EMs is closely related to the immune regulation disorders in patients. ERS exhibits immunosuppressive effects, resulting in greater tumorigenic potential of malignant cells in many tumors. Therefore, it is necessary to investigate the relationship between EMs, ERS, and immune cell infiltration.

In this study, we investigated the roles of ERS‐DEG, *HSPA5, HSP90B1, PDIA6*, and *HERPUD1*, in EMs. These heat shock proteins and ER chaperones were upregulated in ectopic tissues, indicating a potential role in enhancing cell survival and apoptosis resistance in EMs. *HSPA5* and *HSP90B1* are integral to protein folding, shielding cells from ERS‐induced apoptosis [26]. Their overexpression in EMs suggests a mechanism that bolsters cell survival and counteracts apoptosis [27]. The role of *PDIA6* in disulfide bond formation is critical, especially under ERS, where its expression can influence endometrial cell viability by affecting protein conformation and function [28]. The link of *HERPUD1* between ERS and apoptosis indicates its regulatory function in endometrial cell sensitivity to ERS‐induced death, potentially affecting EMs progression [29]. Our study identified ERS‐DEGs that were upregulated in EMs and enhanced cell survival and apoptosis resistance through protein folding and degradation pathways.

We discovered that HUB genes interact with critical pathways such as MAPK, JAK‐STAT, TP53‐regulated metabolism, Wnt signaling, and NF‐κB, which are vital for processes like signal transduction and cell cycle control. *HSPA5* and *HSP90B1* are suggested to modulate the MAPK pathway, influencing endometrial cell behavior and survival in EMs [30]. The JAK‐STAT pathway, which is crucial for immune cell function, may be affected by *PDIA* family proteins and *XBP1*, with *PDIA6* potentially influencing the immune microenvironment of EMs [31]. *HERPUD1* affects TP53 activity and endometrial cell metabolism in EMs [32]. *HSP90B1* may influence EMs development by stabilizing Wnt pathway components [33]. Furthermore, the interaction of *HERPUD1* with NF‐κB might regulate inflammation and immune function in EMs, contributing to chronic inflammation [34]. Our study showed that HUB genes interact with critical pathways in EMs, potentially affecting signal transduction, cell cycle control, and immune modulation.

We identified miRNAs and TFs that regulate genes involved in EMs pathogenesis, including those involved in cell proliferation and immune responses. miRNAs are small non‐coding RNA molecules that regulate gene expression at the post‐transcriptional level by binding to the 3ʹ‐untranslated regions of mRNA. In recent years, the role of miRNAs in EMs has attracted increasing attention, and there is evidence that miRNA dysregulation is closely related to the pathogenesis of EMs. miRNAs can affect various BPs, including cell proliferation, migration, invasion, and apoptosis, which are crucial in the development of EMs. For example, miR‐145 is differentially expressed in EMs and regulates the expression of adhesion molecules and cytoskeletal elements, such as plasminogen activator inhibitor‐1, octamer‐bound TF 4, and myosin‐1, to influence the formation of ectopic lesions. Furthermore, miR‐20a is upregulated in EMs tissues and is associated with disease relapse, suggesting its potential as a biomarker for early detection and relapse surveillance.

Notably, we established a HUB gene‐centered regulatory network and found *ATF4, EP300*, and *NRF1* as TFs for *HSPA5*. Previous studies have demonstrated that *EP300‐mediated HSPA5* acetylation at K353 enhances resistance in pancreatic cancer cells [35], whereas dihydroartemisinin‐induced ERS mitigates iron death [36] in glioma cells through the *PERK/ATF 4/HSPA5* pathway. *HSP90B1* is directly regulated by miR‐223, a mechanism that affects the progression of diseases such as chronic myeloid leukemia [37]. Recent research has indicated that *lncRNA DLX6‐AS1* affects cell growth and invasion in bladder cancer cells through the *miR‐223‐HSP90B1* axis [38]. These findings are consistent with our results. In endometrial cancer, *PDIA6* influences malignant behavior through the *TGF‐β* pathway and its interactions with the *TRPM2‐AS/miR‐424‐5p* axis. Given the hormonal characteristics of EMs and its association with the Wnt pathway [39, 40], *PDIA6* has emerged as a promising regulator of EMs [41]. Our study also suggested an elevation of *HERPID1* expression in the disease, which may contribute to disease progression through its impact on miR‐384. Our study uncovered regulatory mechanisms involving miRNAs, TFs, and key genes in EMs pathogenesis that affect cell proliferation, immune responses, and disease progression.

By analyzing the interactions between ERs‐DEGs and immune cells in EMs, we found significant differences in immune cell abundance between normal and EMs tissues, with T, B, and NK cells influencing the inflammatory environment in EMs [42]. This immune shift may suppress the response to ectopic tissues and promote survival. Core genes involved in cellular stress responses, such as *HSPA5* and *HSP90B1*, may regulate immune responses and potentially affect the infiltration and function of immune cells in EMs. Proteins, such as *PDIA3*, *PDIA6*, and *DNAJC3*, which are involved in protein folding and ERAD, may reflect the efforts of immune cells to manage ERS during inflammation. Furthermore, *MAN*F and *HERPUD*1, which are associated with ERS and UPR, may influence immune cell survival in the chronic inflammatory environment of EMs. The presence of effector memory CD8 T cells may indicate an active cytotoxic response, whereas their accumulation may signify unresolved inflammation. Increased infiltration of mast cells in EMs may promote disease formation by releasing angiogenic and fibrotic factors [43]. The involvement of natural killer T cells may indicate an intrinsic immune dysregulation associated with the immunosuppressive phenotype of EMs. The increase in regulatory T cell frequency suggests a potential immunosuppressive mechanism that protects ectopic tissues while suppressing inflammation [44]. Our analysis of ERS‐DEG interactions with immune cells in EMs revealed significant differences in immune cell abundance, with T, B, and NK cells influencing the inflammatory environment and potentially affecting immune responses and cell survival.

Using GSEA, we identified HUB genes, including *DNAJC10, HSPA5*, and *XBP1*, involved in the ERS response pathway. The expression levels of these genes in immune cells may significantly affect their function in immune responses, particularly in regulating cell survival and function during ERS. Additionally, GO and KEGG pathway analyses revealed significant enrichment of key genes such as *PDIA3* and *PIK3R1* in protein processing, ER‐related signaling pathways, and redox reactions. The high expression of these genes in immune cells may be related to the regulation of their activity during inflammation. Specifically, involvement of *PDIA3* in protein folding and ERS suggests that it may affect cell survival and function by regulating immune cell responses to oxidative stress. By integrating the correlation maps of HUB genes with immune cells, we also identified core genes such as *THBS1* and *PIK3R1*, which exhibited a high degree of correlation with various types of immune cells. These genes not only participate in the regulation of signaling pathways but may also affect the functions of specific immune cells, such as their roles in the TGF‐β signaling and PI3K/AKT pathways, potentially regulating cellular proliferation, differentiation, and the ability to respond to and repair damage. These findings provide a new perspective for understanding the regulatory mechanisms of immune cell function and offer potential targets for the study and treatment of related diseases.

In this study, we focused on the clinical relevance of ERS‐DEGs in EMs. Notably, the upregulation of genes such as *HSPA5, HSP90B1, PDIA6*, and *HERPUD1* correlated closely with the clinical features of EMs, suggesting their potential as biomarkers for EMs diagnosis and prognosis. Furthermore, the interaction of these HUB genes with key signaling pathways like MAPK, JAK‐STAT, TP53, Wnt, and NF‐κB offers new targets for EMs therapy. Our findings underscore the potential of these genes as biomarkers and therapeutic targets, paving the way for future clinical applications, promising diagnostic tools, and effective treatment strategies for patients with EMs.

Although our study provided preliminary evidence for the role of ERS in EMs, it had several limitations. First, this study was mainly based on bioinformatics analysis, which requires further validation of HUB gene expression and function using experimental methods. Second, the limited sample size may affect the generalizability of the results; future studies should expand the sample size to enhance the reliability of the conclusions. Furthermore, in future studies, we plan to explore the functional role of ERS‐DEGs in the development of EMs and their molecular mechanisms, as well as their interactions with immune cell infiltration.

In summary, five diagnostic biomarkers (*HSPA5, HSP90B1, DNAJC3, PDIA6*, and *HERPUD1*) were identified in EMs, providing new perspectives for the diagnosis, mechanism, investigation, and management of EMs.

## Supporting information



Supporting Information

## Data Availability

The data that support the findings of this study are available on request from the corresponding author. The data are not publicly available due to privacy or ethical restrictions.
