# Supplementary material for: Deciphering the Molecular Crosstalk of Endoplasmic Reticulum Stress and Immune Infiltration in Endometriosis
Source: Am J Reprod Immunol. 2025 May 15;93(5):e70092. doi: 10.1111/aji.70092 (PMC12079719; doi:10.1111/aji.70092)
Supplement: Supplementary file 1 — Supporting Information [file AJI-93-e70092-s001.docx]

### Table S1. Endometriosis dataset information list.

|  | GSE120103 | GSE25628 |
| --- | --- | --- |
| Platform | [GPL6480](https://www.ncbi.nlm.nih.gov/geo/query/acc.cgi?acc=GPL6480) | [GPL571](https://www.ncbi.nlm.nih.gov/geo/query/acc.cgi?acc=GPL571) |
| Species | Homo sapiens | Homo sapiens |
| Tissue | endometrium | endometrium |
| Samples in Endometriosis group | 18 | 7 |
| Samples in Normal group | 18 | 6 |

### Table S2.Endoplasmic reticulum stress related genes.

| ERGs | | | | | | | |
| --- | --- | --- | --- | --- | --- | --- | --- |
| ACADVL | BRSK2 | DNAJB2 | FICD | MAP3K5 | PSMC6 | SSR1 | TXNDC12 |
| ADD1 | CALR | DNAJB9 | FKBP14 | MBTPS1 | PTPN1 | STC2 | UBA5 |
| AGR2 | CASP4 | DNAJC10 | FLOT1 | MBTPS2 | PTPN2 | STT3B | UBAC2 |
| AIFM1 | CAV1 | DNAJC3 | FOXRED2 | NCK1 | RASGRF1 | STUB1 | UBE2G2 |
| ALOX15 | CCDC47 | EDEM1 | GET4 | NCK2 | RASGRF2 | SULT1A3 | UBE2J1 |
| AMFR | CCL2 | EDEM2 | GFPT1 | NFE2L1 | RHBDD1 | SVIP | UBE2J2 |
| ANKS4B | CCND1 | EDEM3 | GORASP2 | NFE2L2 | RNF121 | SYVN1 | UBE2K |
| ANKZF1 | CDK5RAP3 | EEF2 | GOSR2 | NPLOC4 | RNF139 | TARDBP | UBE4A |
| APAF1 | CEBPB | EIF2AK2 | GRINA | NRBF2 | RNF183 | TATDN2 | UBE4B |
| ARFGAP1 | CFTR | EIF2AK3 | GSK3A | OPA1 | RNF185 | TBL2 | UBQLN1 |
| ASNS | CHAC1 | EIF2AK4 | GSK3B | OS9 | RNF186 | THBS1 | UBQLN2 |
| ATF3 | CLU | EIF2B5 | HDGF | P4HB | RNF5 | THBS4 | UBXN1 |
| ATF4 | COPS5 | EIF2S1 | HERPUD1 | PARK7 | RNFT1 | TLN1 | UBXN4 |
| ATF6B | CREB3L1 | EP300 | HM13 | PARP16 | SCAMP5 | TMBIM6 | UBXN6 |
| ATF6 | CREB3L2 | ERLEC1 | HSP90B1 | PDIA2 | SDF2L1 | TMCO1 | UBXN8 |
| ATG10 | CREB3L3 | ERLIN1 | HSPA13 | PDIA3 | SDF2 | TMEM117 | UFC1 |
| ATP2A1 | CREB3L4 | ERLIN2 | HSPA1A | PDIA4 | SEC16A | TMEM33 | UFM1 |
| ATP2A2 | CREB3 | ERN1 | HSPA5 | PDIA5 | SEC31A | TMEM67 | UGGT1 |
| ATP6V0D1 | CTH | ERN2 | HYOU1 | PDIA6 | SEC61B | TMTC3 | UGGT2 |
| ATXN3 | CXXC1 | ERP27 | IGFBP1 | PDX1 | SEL1L | TMUB1 | USP13 |
| AUP1 | DAB2IP | ERP29 | ITPR1 | PIK3R1 | SERINC3 | TMX1 | USP14 |
| BAK1 | DCTN1 | ERP44 | JKAMP | PIK3R2 | SERP1 | TNFRSF10B | USP19 |
| BAX | DDIT3 | EXTL1 | JUN | PLA2G6 | SERP2 | TOR1A | USP25 |
| BBC3 | DDRGK1 | EXTL2 | KDELR3 | PMAIP1 | SESN2 | TP53 | VAPB |
| BCAP31 | DERL1 | EXTL3 | KLHDC3 | PML | SGTA | TPP1 | VCP |
| BCL2L11 | DERL2 | FAF2 | LMNA | POMT2 | SGTB | TRAF2 | WFS1 |
| BCL2L1 | DERL3 | FBXO2 | LRRK2 | PPP1R15A | SHC1 | TRIB3 | WIPI1 |
| BCL2 | DNAJB11 | FBXO6 | MAGEA3 | PPP1R15B | SIRT1 | TRIM13 | XBP1 |
| BFAR | DNAJB12 | FCGR2B | MAN1B1 | PPP2CB | SRPRB | TRIM25 | YIF1A |
| BOK | DNAJB14 | FGF21 | MANF | PREB | SRPX | TTC23L | YOD1 |

### Table S3. GO enrichment analysis results of ERGSDEGs.

| ONTOLOGY | ID | Description | GeneRatio | BgRatio | p.adjust | qvalue |
| --- | --- | --- | --- | --- | --- | --- |
| BP | GO:0034976 | response to endoplasmic reticulum stress | 29/33 | 257/18800 | 8.84 e-48 | 5.41 e-48 |
| BP | GO:0035966 | response to topologically incorrect protein | 18/33 | 160/18800 | 1.26 e-26 | 7.71 e-27 |
| BP | GO:0006986 | response to unfolded protein | 17/33 | 139/18800 | 9.98 e-26 | 6.1 e-26 |
| BP | GO:0035967 | cellular response to topologically incorrect protein | 13/33 | 117/18800 | 1.8 e-18 | 1.1 e-18 |
| CC | GO:0005788 | endoplasmic reticulum lumen | 11/33 | 311/19594 | 1.76 e-10 | 1.21 e-10 |
| CC | GO:0034663 | endoplasmic reticulum chaperone complex | 5/33 | 11/19594 | 2.08 e-10 | 1.43 e-10 |
| CC | GO:0140534 | endoplasmic reticulum protein-containing complex | 7/33 | 125/19594 | 4.14 e-08 | 2.84 e-08 |
| CC | GO:0016529 | sarcoplasmic reticulum | 6/33 | 71/19594 | 4.3 e-08 | 2.95 e-08 |
| MF | GO:0015036 | disulfide oxidoreductase activity | 5/33 | 42/18410 | 2.02 e-06 | 1.36 e-06 |
| MF | GO:0016667 | oxidoreductase activity, acting on a sulfur group of donors | 5/33 | 57/18410 | 4.89 e-06 | 3.28 e-06 |
| MF | GO:0051787 | misfolded protein binding | 4/33 | 29/18410 | 1.21 e-05 | 8.15 e-06 |
| MF | GO:0015035 | protein-disulfide reductase activity | 4/33 | 37/18410 | 2.51 e-05 | 1.68 e-05 |

GO: Gene Ontology; BP: biological process; CC: cellular component; MF: molecular function; ERGSDEGs: Endoplasmic reticulum stress related genes.

### Table S4. KEGG enrichment analysis results of ERGSDEGs.

| ONTOLOGY | ID | Description | GeneRatio | BgRatio | p.adjust | qvalue |
| --- | --- | --- | --- | --- | --- | --- |
| KEGG | hsa04141 | Protein processing in endoplasmic reticulum | 14/25 | 171/8164 | 1.12 e-15 | 6.83 e-16 |
| KEGG | hsa05417 | Lipid and atherosclerosis | 6/25 | 215/8164 | 0.003043 | 0.00185 |
| KEGG | hsa04918 | Thyroid hormone synthesis | 4/25 | 75/8164 | 0.004021 | 0.002444 |
| KEGG | hsa05164 | Influenza A | 5/25 | 171/8164 | 0.00604 | 0.003671 |
| KEGG | hsa05215 | Prostate cancer | 4/25 | 97/8164 | 0.006573 | 0.003995 |
| KEGG | hsa05167 | Kaposi sarcoma-associated herpesvirus infection | 5/25 | 194/8164 | 0.007268 | 0.004417 |
| KEGG | hsa04510 | Focal adhesion | 5/25 | 201/8164 | 0.007345 | 0.004464 |
| KEGG | hsa05165 | Human papillomavirus infection | 6/25 | 331/8164 | 0.007668 | 0.004661 |
| KEGG | hsa05144 | Malaria | 3/25 | 50/8164 | 0.007668 | 0.004661 |
| KEGG | hsa04919 | Thyroid hormone signaling pathway | 4/25 | 121/8164 | 0.007668 | 0.004661 |
| KEGG | hsa05163 | Human cytomegalovirus infection | 5/25 | 225/8164 | 0.00786 | 0.004777 |
| KEGG | hsa05162 | Measles | 4/25 | 139/8164 | 0.010793 | 0.00656 |

KEGG, Kyoto Encyclopedia of Genes and Genomes; ERGSDEGs: Endoplasmic reticulum stress related genes.

### Table S5. GSEA enrichment analysis results of ERGSDEGs.

| ID | setSize | enrichmentScore | NES | pvalue | p.adjust | qvalue |
| --- | --- | --- | --- | --- | --- | --- |
| KEGG_MAPK_SIGNALING_PATHWAY | 225 | 0.35839511 | 1.72303985 | 2.3685 e-05 | 0.00024555 | 0.00016603 |
| KEGG_JAK_STAT_SIGNALING_PATHWAY | 129 | 0.40400818 | 1.8032125 | 7.2723 e-05 | 0.00064787 | 0.00043806 |
| REACTOME_TP53_REGULATES_METABOLIC_GENES | 57 | 0.47970018 | 1.94699273 | 0.00012923 | 0.00103695 | 0.00070113 |
| WNT_SIGNALING | 75 | 0.31011071 | 1.32112049 | 0.06666667 | 0.16904762 | 0.1143009 |
| REACTOME_NF_KB_ACTIVATION_THROUGH_FADD_RIP_1_PATHWAY_MEDIATED_BY_CASPASE_8_AND_10 | 10 | 0.57685143 | 1.44901759 | 0.07218684 | 0.17784833 | 0.12025147 |
| KEGG_NEUROACTIVE_LIGAND_RECEPTOR_INTERACTION | 231 | 0.53361727 | 2.57235531 | 1E-10 | 3.2096 e-09 | 2.1702 e-09 |
| KEGG_CYTOKINE_CYTOKINE_RECEPTOR_INTERACTION | 215 | 0.52484725 | 2.52154228 | 1E-10 | 3.2096 e-09 | 2.1702 e-09 |
| WP_GPCRS_CLASS_A_RHODOPSINLIKE | 199 | 0.52835244 | 2.51475389 | 1E-10 | 3.2096 e-09 | 2.1702 e-09 |
| NABA_SECRETED_FACTORS | 240 | 0.51303732 | 2.49207464 | 1E-10 | 3.2096 e-09 | 2.1702 e-09 |
| WP_OVERVIEW_OF_PROINFLAMMATORY_AND_PROFIBROTIC_MEDIATORS | 91 | 0.57803578 | 2.44957997 | 1E-10 | 3.2096 e-09 | 2.1702 e-09 |
| REACTOME_SRP_DEPENDENT_COTRANSLATIONAL_PROTEIN_TARGETING_TO_MEMBRANE | 86 | 0.80375398 | 3.49843032 | 1E-10 | 3.2096 e-09 | 2.1702 e-09 |
| REACTOME_EUKARYOTIC_TRANSLATION_INITIATION | 93 | 0.76461641 | 3.42883905 | 1E-10 | 3.2096 e-09 | 2.1702 e-09 |
| REACTOME_SELENOAMINO_ACID_METABOLISM | 81 | 0.7804899 | 3.38849082 | 1E-10 | 3.2096 e-09 | 2.1702 e-09 |
| REACTOME_NONSENSE_MEDIATED_DECAY_NMD | 89 | 0.76200014 | 3.36367694 | 1E-10 | 3.2096 e-09 | 2.1702 e-09 |
| WP_CYTOPLASMIC_RIBOSOMAL_PROTEINS | 67 | 0.81317332 | 3.36342113 | 1E-10 | 3.2096 e-09 | 2.1702 e-09 |

### Table S6. Description of hub genes.

| Gene Symbol | Description | Category | Uniprot ID | Gifts | GC Id | Relevance score |
| --- | --- | --- | --- | --- | --- | --- |
| XBP1 | X-Box Binding Protein 1 | Protein Coding | P17861 | 49 | GC22M028794 | 77.7571 |
| HSPA5 | Heat Shock Protein Family A (Hsp70) Member 5 | Protein Coding | P11021 | 52 | GC09M125234 | 72.36229 |
| DNAJC3 | DnaJ Heat Shock Protein Family (Hsp40) Member C3 | Protein Coding | Q13217 | 44 | GC13P095677 | 50.45953 |
| MANF | Mesencephalic Astrocyte Derived Neurotrophic Factor | Protein Coding | P55145 | 43 | GC03P051385 | 44.63544 |
| HERPUD1 | Homocysteine Inducible ER Protein With Ubiquitin Like Domain 1 | Protein Coding | Q15011 | 42 | GC16P056932 | 37.50907 |
| PDIA6 | Protein Disulfide Isomerase Family A Member 6 | Protein Coding | Q15084 | 42 | GC02M010784 | 53.11225 |
| PDIA3 | Protein Disulfide Isomerase Family A Member 3 | Protein Coding | P30101 | 47 | GC15P043746 | 48.39243 |
| PDIA4 | Protein Disulfide Isomerase Family A Member 4 | Protein Coding | P13667 | 45 | GC07M149003 | 44.59092 |
| HSP90B1 | Heat Shock Protein 90 Beta Family Member 1 | Protein Coding | P14625 | 51 | GC12P103930 | 41.74989 |

### Table S7. mRNA-miRNA

| mRNA | miRNA |
| --- | --- |
| HSPA5 | hsa-miR-3121-3p |
| HSPA5 | hsa-miR-5688 |
| HSPA5 | hsa-miR-635 |
| HSPA5 | hsa-miR-6774-5p |
| HSPA5 | hsa-miR-495-3p |
| HSPA5 | hsa-miR-338-5p |
| HSPA5 | hsa-miR-7-2-3p |
| HSPA5 | hsa-miR-7-1-3p |
| XBP1 | hsa-miR-5585-5p |
| XBP1 | hsa-miR-3119 |
| XBP1 | hsa-miR-4999-3p |
| XBP1 | hsa-miR-181b-3p |
| XBP1 | hsa-miR-181b-2-3p |
| XBP1 | hsa-miR-4420 |
| HSP90B1 | hsa-miR-1-3p |
| HSP90B1 | hsa-miR-613 |
| HSP90B1 | hsa-miR-206 |
| HSP90B1 | hsa-miR-4643 |
| HSP90B1 | hsa-miR-223-3p |
| HSP90B1 | hsa-miR-450b-3p |
| HSP90B1 | hsa-miR-4789-5p |
| HSP90B1 | hsa-miR-4262 |
| HSP90B1 | hsa-miR-769-3p |
| HSP90B1 | hsa-miR-181c-5p |
| HSP90B1 | hsa-miR-181d-5p |
| HSP90B1 | hsa-miR-181a-5p |
| HSP90B1 | hsa-miR-181b-5p |
| DNAJC3 | hsa-miR-335-3p |
| DNAJC3 | hsa-miR-200c-3p |
| DNAJC3 | hsa-miR-429 |
| DNAJC3 | hsa-miR-200b-3p |
| DNAJC3 | hsa-miR-607 |
| DNAJC3 | hsa-miR-4753-3p |
| DNAJC3 | hsa-miR-4262 |
| DNAJC3 | hsa-miR-6778-3p |
| PDIA3 | hsa-let-7c-3p |
| PDIA3 | hsa-miR-1303 |
| PDIA3 | hsa-miR-3613-3p |
| PDIA3 | hsa-miR-4303 |
| PDIA3 | hsa-miR-4456 |
| PDIA3 | hsa-miR-1287-3p |
| PDIA6 | hsa-miR-23a-3p |
| PDIA6 | hsa-miR-23c |
| PDIA6 | hsa-miR-23b-3p |
| PDIA6 | hsa-miR-654-5p |
| PDIA6 | hsa-miR-541-3p |
| PDIA6 | hsa-miR-376a-3p |
| PDIA6 | hsa-miR-376b-3p |
| PDIA6 | hsa-miR-4708-5p |
| PDIA6 | hsa-miR-526b-5p |
| PDIA6 | hsa-miR-3148 |
| PDIA6 | hsa-miR-6814-5p |
| PDIA6 | hsa-miR-8063 |
| PDIA6 | hsa-miR-4698 |
| PDIA6 | hsa-miR-6768-3p |
| PDIA6 | hsa-miR-676-5p |
| PDIA4 | hsa-miR-539-5p |
| PDIA4 | hsa-miR-892c-5p |
| HERPUD1 | hsa-miR-548e-5p |
| HERPUD1 | hsa-miR-9-3p |
| HERPUD1 | hsa-miR-6796-3p |
| HERPUD1 | hsa-miR-384 |

### Table S8. mRNA-TF

| mRNA | Transcription factor |
| --- | --- |
| DNAJC3 | NRF1 |
| DNAJC3 | SPI1 |
| HERPUD1 | MAX |
| HERPUD1 | MNT |
| HERPUD1 | MYC |
| HERPUD1 | SPI1 |
| HERPUD1 | USF1 |
| HERPUD1 | USF2 |
| HERPUD1 | YY1 |
| HERPUD1 | ATF4 |
| HERPUD1 | BHLHE40 |
| HERPUD1 | CEBPB |
| HERPUD1 | FOXA1 |
| HERPUD1 | FOXA2 |
| HSP90B1 | ELF1 |
| HSP90B1 | GABPA |
| HSP90B1 | MAX |
| HSP90B1 | SPI1 |
| HSPA5 | ATF4 |
| HSPA5 | CEBPB |
| HSPA5 | CREB1 |
| HSPA5 | CTCF |
| HSPA5 | ELF1 |
| HSPA5 | EP300 |
| HSPA5 | ERG |
| HSPA5 | ETS1 |
| HSPA5 | FOS |
| HSPA5 | FOSL1 |
| HSPA5 | FOSL2 |
| HSPA5 | GABPA |
| HSPA5 | JUN |
| HSPA5 | JUND |
| HSPA5 | MAX |
| HSPA5 | MYC |
| HSPA5 | NR3C1 |
| HSPA5 | NRF1 |
| HSPA5 | POLR2A |
| HSPA5 | REST |
| HSPA5 | RUNX1 |
| HSPA5 | SMARCA4 |
| HSPA5 | SMARCB1 |
| HSPA5 | SMARCC1 |
| HSPA5 | STAT3 |
| HSPA5 | TBP |
| HSPA5 | YY1 |
| MANF | ELF1 |
| MANF | ERG |
| MANF | GABPA |
| MANF | YY1 |
| PDIA4 | NRF1 |
| PDIA4 | SPI1 |
| PDIA4 | EGR1 |
| PDIA4 | ELF1 |
| PDIA4 | ERG |
| PDIA4 | FOS |
| PDIA4 | GABPA |
| PDIA4 | MAX |
| PDIA4 | MYC |
| PDIA6 | CTCF |
| PDIA6 | EGR1 |
| PDIA6 | ELF1 |
| PDIA6 | ERG |
| PDIA6 | FOXA1 |
| PDIA6 | GABPA |
| PDIA6 | NRF1 |
| PDIA6 | RAD21 |
| PDIA6 | SMC3 |
| PDIA6 | STAG1 |
| XBP1 | EP300 |
| XBP1 | ERG |
| XBP1 | ESR1 |
| XBP1 | FOXA1 |
| XBP1 | FOXA2 |
| XBP1 | HOXB13 |
| XBP1 | MAX |
| XBP1 | MYC |
| XBP1 | RAD21 |
| XBP1 | RUNX1 |
| XBP1 | SMC3 |
| XBP1 | SPI1 |
| XBP1 | STAG1 |
| XBP1 | TCF3 |
| XBP1 | CEBPA |
| XBP1 | VDR |
| XBP1 | CEBPB |
| XBP1 | AR |
| XBP1 | CTCF |
